# Plant-Derived Protectants in Combating Soil-Borne Fungal Infections in Tomato and Chilli

**DOI:** 10.3390/jof8020213

**Published:** 2022-02-21

**Authors:** Himanshu Arora, Abhishek Sharma, Peter Poczai, Satyawati Sharma, Farah Farhanah Haron, Abdul Gafur, R. Z. Sayyed

**Affiliations:** 1 Centre for Rural Development and Technology, Indian Institute of Technology, New Delhi 110016, India; himanshuarora592@gmail.com (H.A.); satyawatis@hotmail.com (S.S.); 2 Amity Food and Agriculture Foundation, Amity University, Noida 201313, India; 3 Botany Unit, Finnish Museum of Natural History, University of Helsinki, P.O. Box 7, FI-00014 Helsinki, Finland; 4 Biological Control Programme, Agrobiodiversity and Environment Research Centre, Malaysian Agricultural Research and Development Institute, Serdang 43400, Malaysia; farahfarhanah@mardi.gov.my; 5 Sinarmas Forestry Corporate Research and Development, Perawang 28772, Indonesia; gafur@uwalumni.com; 6 Department of Microbiology, PSGVP Mandal’s Arts, Science, and Commerce College, Shahada 425409, India; sayyedrz@gmail.com

**Keywords:** plant secondary metabolites, botanical pesticides, plant diseases, essential oil, soil amendments, in silico

## Abstract

Fungal infections transmitted through the soil continue to pose a threat to a variety of horticultural and agricultural products, including tomato and chilli. The indiscriminate use of synthetic pesticides has resulted in a slew of unintended consequences for the surrounding ecosystem. To achieve sustainable productivity, experts have turned their attention to natural alternatives. Due to their biodegradability, varied mode of action, and minimal toxicity to non-target organisms, plant-derived protectants (PDPs) are being hailed as a superior replacement for plant pesticides. This review outlines PDPs’ critical functions (including formulations) in regulating soil-borne fungal diseases, keeping tomato and chilli pathogens in the spotlight. An in-depth examination of the impact of PDPs on pathogen activity will be a priority. Additionally, this review emphasises the advantages of the in silico approach over conventional approaches for screening plants’ secondary metabolites with target-specific fungicidal activity. Despite the recent advances in our understanding of the fungicidal capabilities of various PDPs, it is taking much longer for that information to be applied to commercially available pesticides. The restrictions to solving this issue can be lifted by breakthroughs in formulation technology, governmental support, and a willingness to pursue green alternatives among farmers and industries.

## 1. Introduction

Synthetic pesticides have long been used to effectively manage plant diseases. However, their prolonged and persistent use has resulted in many detrimental and unprecedented effects on the surrounding environment. Pesticide misuse has resulted in many phytopathogens becoming resistant [1]. Pesticides’ bioaccumulation and toxicity to non-target organisms have also had negative environmental repercussions [2]. Synthetic pesticides cause nearly two lakhs of deaths from poisoning each year, and 99% of them occur in developing countries [3]. Although synthetic pesticides are sometimes more convenient, biopesticides derived from natural resources are a superior option. This last decade has seen the various botanicals becoming increasingly prominent in the field of plant protection.

**Figure 1:**
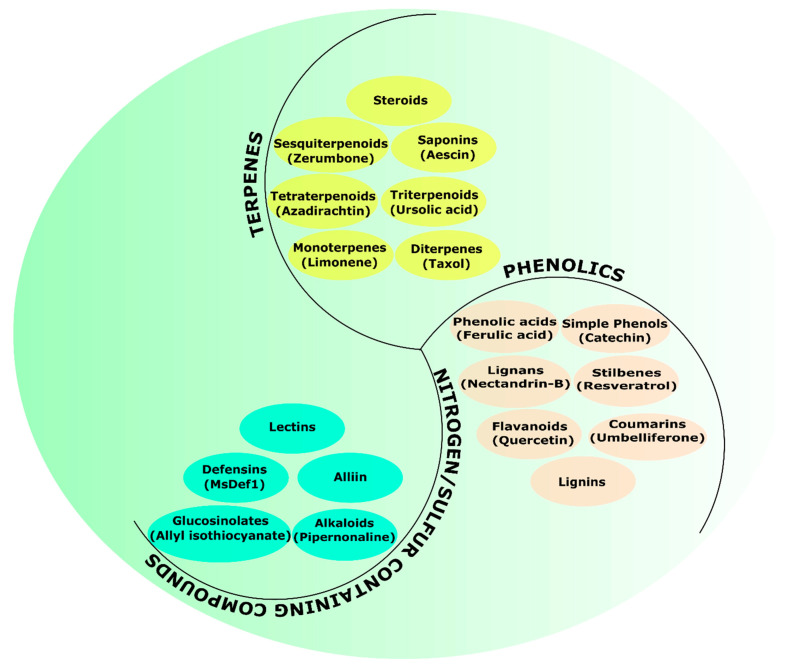
Types of secondary metabolites in plants.

Plant-derived (botanical) protectants (PDPs) provide a competitive advantage over synthetic pesticides because they are safer and cheaper. We employ the term “plant-derived protectants” throughout this article to refer to purified active metabolites, crude plant extracts, essential oils, and total phytobiomass. They have multiple modes of action, are biodegradable, and reduced non-target toxicity [4,5,6]. Regardless of the form of PDPs used, their pesticidal activity is mainly due to bioactive secondary metabolites. Secondary metabolite synthesis is part of the plant’s defense strategy. These metabolites might play a crucial role in the health of the plant, but they predominantly act as plant defense agents [7]. In accordance with the biosynthetic principle, plant secondary metabolites can be divided into three main classifications, which are terpenes, phenolics, and nitrogen-containing chemicals (Figure 1) [8]. Terpenes are the most abundant and significant class of secondary metabolites. Monoterpenes are found in abundance in essential oils, which account for about 80% of the total [9]. Phenols are a class of chemical compounds with a wide range of sizes and miscibility in water and organic solvents. The single-substituted phenolic rings are typically found in simple phenolics, while the complex compounds have phenolic rings connected to several functional groups. Nitrogen-containing secondary metabolites are usually biosynthesised amino acid derivatives.

According to the Food and Agriculture Organization (FAO, 2019), worldwide production of tomatoes was almost 180 million tonnes, with dry chiles and peppers at over 4 million tonnes. Unfortunately, tomato and chilli fungal infections have held down market demand for these products. These infections are a hidden, but frequent, roadblock in making tomato and chilli crops profitable. Diseases caused by soil-borne fungal phytopathogens such as *Fusarium* spp., *Rhizoctonia* spp., *Phytophthora* spp., *Pythium* spp., *Sclerotinia* spp., and *Verticillium* spp. have resulted in substantial yield losses in these two crops [10,11,12,13,14]. In tomato production, vascular wilt induced by *Fusarium* spp. involves infection of plants’ roots, and once this is complete, the vascular system attacks accelerate, leading plants to wilt and lose 10–80% of their yield [14]. *Phytophthora capsici*, a fungus that causes root and crown rot in peppers, has led to disease losses of up to 40% [13]. As with damping-off, *Pythium* species induce premature death of chilli seedlings in nurseries and greenhouses [10].

PDPs could be a promising tool to regulate soil-borne fungal diseases in *Solanum lycopersicum* (Tomato) and *Capsicum annuum* (chilli). Hence, this analysis focuses on every facet of scientific development, starting with screening PDPs, to assessing their application possibilities regarding ways of functioning against fungal phytopathogens of tomatoes and chilli. Additionally, this assessment throws light on enigmas in the process of successfully commercialising scientific endeavors.

## 2. Management of Soil-Borne Diseases in Tomato and Chilli

### 2.1. Using Crude Plant Extracts, Essential Oils, and Purified Secondary Metabolites (Lab-to-Land Approach)

Applying botanicals or their metabolites straight to the field is likely to yield a variable result [15]. To circumvent this constraint, researchers are designing potent formulations (a combination of one or more active substances and inert components) capable of efficiently managing a pest over an extended period of time in the field [4]. Such “lab to land” strategies boost farmers’ abilities to combat crop disease and add economic and societal value to research. Contrary to this fact, we continue to see research using unformulated PDPs, even for field evaluations of disease control.

More than any other soil-borne pathogen in tomatoes, *Fusarium* management has been studied extensively (Table 1). Purified pomegranate peel water extract (Pae) inhibited mycelial development of *F. oxysporum* f. sp. *lycopersici* by 83% at a concentration of 0.5% (*w*/*v*) with higher concentrations of phenolic acid-like punicalagins and ellagic acid [16]. When employed in soil treatment, pure Pae was likewise effective at reducing disease incidences in treated tomato plants to about 58% in treatment when compared to 100% in the untreated control. Among others, extracts of *Allium tuncelianum*, *A. sativum*, *Azadirachta indica*, *Zingiber officinale*, *Pistacia lentiscus*, *Moringa oleifera*, *Stevia rebaudiana*, *Theobroma cacao*, *Juglans macrocarpa*, and *J. mollis* successfully inhibited the disease and symptom development in tomato plants against various *Fusarium* species [13,17,18,19,20,21]. Tomato plants cultivated in a non-circulating hydroponic system were treated with *Thymbra capitata* essential oil at 1.473 µL/L concentration, resulting in a 30.76% reduction in disease severity caused by *F. oxysporum* f. sp. *radicis-lycopersi* [22]. A study evaluating various essential oils for antifungal activity against *F. oxysporum* f. sp. *lycopersici* discovered that *Syzygium aromaticum* oil was the most effective. The oil was later utilised as a 5% aqueous emulsion where it exhibited an 86.5% decrease in Fusarium wilt in tomato during a pot trial [23]. A subsequent investigation explored a 5% (*w*/*w*) nanoemulsion formulation that used the previously mentioned *Syzygium aromaticum* and *Cymbopogon citratus* essential oils for synergistic potential. The formulation at 4000 mg/L provided 67.51% wilt disease control [24]. The finding demonstrates that synergy is a novel notion that should be investigated more regularly for the purpose of ensuring sustained plant protection. At a 1.5% ethanol extract concentration, Ozkaya and Ergun [13] found *Allium tuncelianum* to be efficient against *Pythium deliense*, *Rhizoctonia solani*, *Sclerotinia sclerotiorum*, and Sclerotium *rolfsii*. The investigation using *Euphorbia* latex derivatives to control *Verticillium dahliae* in tomatoes found that the seed treatment reduced symptoms significantly [25,26]. Mekam et al. [27] observed that spraying *Euphorbia hirta* leaf ethanolic extract at 2.50 mg/mL offered protection against soil-borne *Rhizoctonia solani*. Table 1 summarises botanicals employed to control soil-borne fungal diseases of tomatoes. 

Though prospective PSMs are infrequently utilised to manage soil-borne infections in chilli plants compared to tomatoes, their application has improved significantly in recent years (Table 2). Wang et al. [44] studied the efficacy of cuminic acid, a pure compound extracted from the seeds of *Cuminum cyminum* against *Phytophthora capsici*. Cuminic acid at 1000 µg/mL concentration proved to be quite efficacious in managing the disease and exhibited 70.89% disease control. Methanolic extracts of *Boerhavia diffusa* roots at 1% concentration significantly reduced the diseases symptoms caused by *Ph. Capsici* in chilli plants [45]. At a concentration of 100 ppm, *Cymbopogon citratus* essential oil demonstrated a 60.5% reduction in disease severity, which confirmed its effectiveness against *Ph. Capsici* [46]. A study by Pandey et al. [47] concluded that aqueous extract of *Lantana camara* leaves is particularly efficient in managing pre- and post-emergence damping-off caused by *P. aphanidermatum*. Seed treatment with the *Glycyrrhiza uralensis* root extracts greatly reduced the seedling mortality caused by *P. aphanidermatum* and *R. solani* [48].

**Figure 2:**
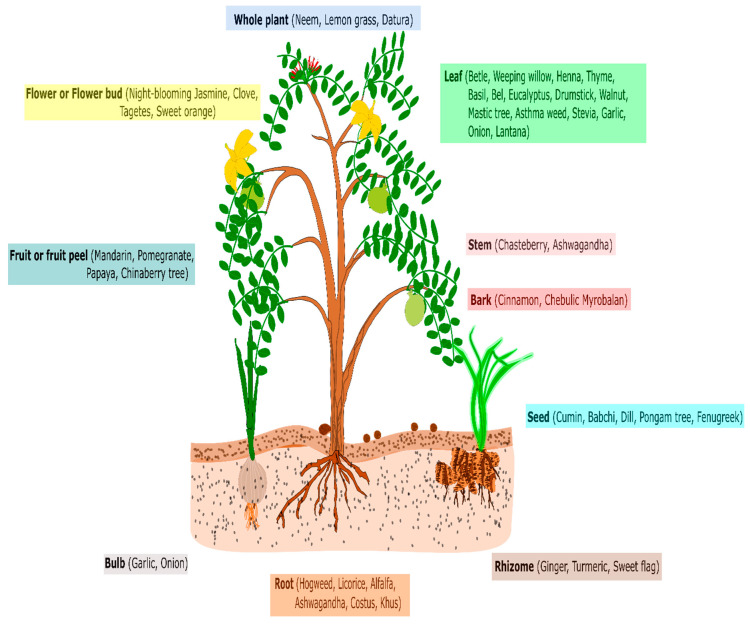
Parts of known plant species rich with bioactive compounds.

The bioactive metabolites in the plant parts of certain species have been discovered to have a significant pesticidal value (Figure 2). While the pesticidal effects of purified metabolites are exploited in a variety of crops [52,53,54], we discovered very little research on tomatoes and chilli peppers. Smaili et al. [25,26], in their study, used derivatives of the α-euphorbol, 31-norlanostenol, and lupeol acetate compounds isolated from the latex of different *Euphorbia* spp. as a seed treatment and spray for the control of *Verticillium dahliae* in tomato, and observed a significant reduction in symptom development at a very low concentration of 10 µg/mL. The derivatives of these compounds worked as the elicitors of plant defense. Wang et al. [44] studied the efficacy of cuminic acid, a pure compound extracted from the seeds of *Cuminum cyminum* against *Phytophthora capsici*. Cuminic acid, other than exhibiting significant inhibition of mycelia and zoospore germination, proved to be quite efficacious at 1000 µg/mL concentration in managing the disease and exhibited 70.89% disease control. Even though purified compounds are highly effective, utilising them is an expensive affair due to the cost of compound isolation/purification and recovery of a minute proportion of the component. 

### 2.2. Using Total Phytobiomass (Land to Land Approach)

Studies on the efficacy of PDPs against phytopathogens primarily aim to develop them as fungicides. Using these bioactive-rich plants as green manure or companion plants provides an efficient alternative, harnessing their allelopathic capabilities. Brassicaceae crops used as soil biofumigant have garnered considerable interest as an alternative to methyl bromide. The hydrolysis of sulfur and nitrogen-containing glucosinolates (GLS) found in plants from the Brassicaceae family into extremely toxic volatile isothiocyanates is triggered by the enzyme myrosinase in the presence of water [55]. In an in-vitro experiment by Pane et al. [56], volatiles obtained from *Brassica carinata* seed meal (BCSM) showed dose-dependent growth inhibition of *F. oxysporum* f. sp. *lycopersici*. Complete growth inhibition was obtained at 100 mg/mL concentration. Also, when combined with thyme oil and *Bacillus amyloliquefaciens*, the volatiles inhibited growth up to 34% more than the bacterial antagonist alone. The use of BCSM at a rate of 0.5 g/pot reduced the incidence and severity of Fusarium wilt in tomato plants by around 50%. According to Ma et al. [57], seed meal amendments affected the fungal mycobiota of the soil. They observed an increase in the *Fusarium*, *Hypocreales*, and *Chaetomium* populations when they employed *Camelina sativa* seed meal as an amendment due to the increased nutrient availability. 

Al-Hammouri et al. [58] found that the root-associated *R. solani* of chilli reduced when they amended the soil with *Calligonum* aboveground plant parts and olive leaves. As described in the literature, the principal active chemicals in olive leaves include phenols such as oleuropein and hydroxytyrosol. Besides, it was thought that the soil microbiome would change for the better and help the chilli plants. Kadoglidou et al. [12] used spearmint and Greek oregano aboveground plant parts as soil amendments at 4% (*w*/*w*) against soil-borne diseases (Fusarium and Vertcillium wilt) of tomato plants. After amendments, GC/MS examination of soil samples revealed a decrease in monoterpenes and an increase in sesquiterpenes. Growing parameters improved due to the long-term persistence of plant-growth-promoting sesquiterpenes, oxygenated monoterpenes such as carvacrol in the soil, along with high microbial activity, due to degradation of plant material. Even after 50 days, the tomato plant showed no signs of Fusarium or Verticillium wilt.

## 3. Antifungal Screening Assays of PDPs

When it comes to evaluating the antifungal potential of PDPs (pure metabolite or in crude form as extracts or essential oils), screening assays are unmatched, as they aid in labelling them as entitled and qualified for further advancement. The following subsections will detail the typical screening procedures utilised and a potential alternative to these conventional approaches.

### 3.1. Conventional Approach

For starting to narrow down possible antifungal PDPs, there are three types of broad classification. This classification includes dilution methods, diffusion methods, and bioautographic procedures. Well-known dilution methods are agar and broth dilutions, wherein the culture medium is mixed with the test sample(s) and inoculated with the target pathogen. Additionally, macro- or micro-dilution techniques aid in determining the minimal inhibitory and fungicidal concentrations of the test sample [59,60]. The diffusion methods involve well diffusion, disc diffusion, and poison food techniques [60]. However, diffusion methods are often inadequate to reveal the actual antifungal activity because of the meager diffusion rate of hydrophobic bioactive metabolites through agar media. In bioautographic methods, the use of thin-layer chromatography (TLC) precedes the antifungal assay. The bioautographic methods can be divided into contact, direct, and immersion bioautography [61]. Despite their widespread use, these conventional procedures frequently fall short due to low reproducibility and other restrictions such as higher cost, sluggishness, and arduousness.

### 3.2. In Silico Approach

**Figure 3:**
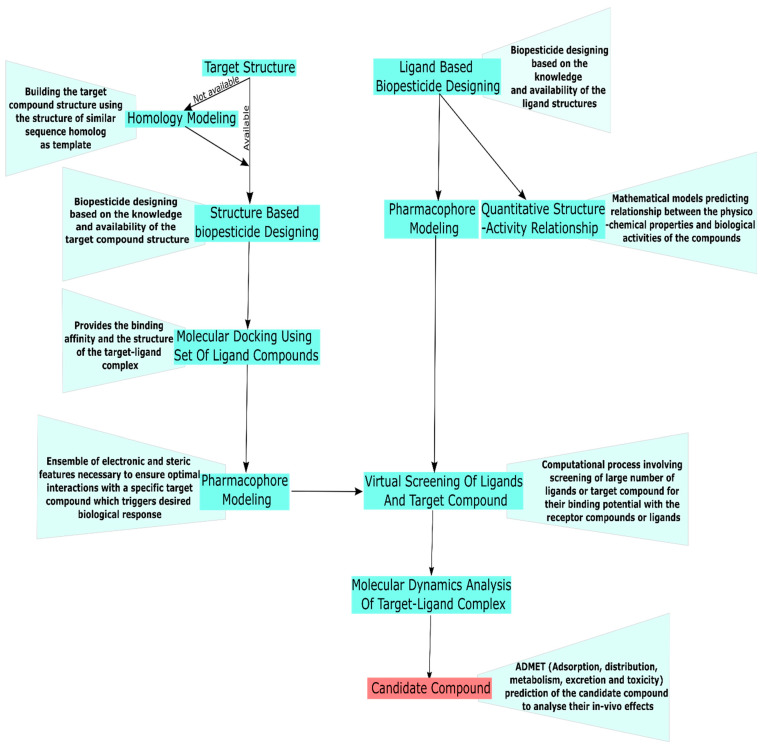
Schematic view of the steps involved in candidate compound identification and design (in silico approach).

Thanks to the development of bioinformatics, scientists can use computer-aided technologies to screen antifungal compounds with knowledge obtained from genomic sequencing of the pathogens and computational work on the structures of antifungal compounds and the biological targets [62,63]. Such an in-silico approach has aided in the rapid screening of several antifungal chemicals. These computationally selected, natural antifungal chemicals rely on their binding affinity with the targeted virulent compounds. Virulence proteins involved in sterol, chitin, melanin, tubulin, and protein biosynthesis have been the target of interest for synthetic fungicides [64]. Researchers have shown interest in targeting virulence proteins linked to sterol, chitin, melanin, and tubulin biosynthesis [64]. Pathogenesis-related genes and their translational products involved in the cell wall or membrane degradation, melanin biosynthesis, phytotoxin synthesis, effector protein synthesis, and fungal cells growth/differentiation have been the major targets for these computational based studies [65,66,67,68]. The different processes of biopesticide design are illustrated in Figure 3. The 3D structure of required binding molecules can be obtained using special databases such as PubChem and ChEMBL [69,70]. 

Silva et al. [71] targeted the β-glucosidases (FsBglc) protein of *F. solani* f. sp. *piperis*, which have a critical role in host cell wall degradation. The 3D structure for the FsBglc protein was prepared using the β-glucosidases enzyme from *Kluyveromyces marxianus* (KmBglI) as a template through homology modelling. The ligand molecules eugenol and methyl eugenol showed negative MolDock scores and H bond energy when interacted with FsBg1c protein. The presence of many hydroxyl groups and a benzene ring in eugenol and methyl eugenol disrupted the hydrogen bonding network, resulting in negative H-bond energy and MolDock scores of these ligand molecules. The in-vitro and in-vivo control of *Alternaria alternata* by *Anadenanthera colubrina* methanol extract, for some part, was attributed to the presence of β-sitosterol and β-sitosteryl linoleate by Campos et al. [72]. Both of these bioactive compounds, which share some structural similarities with ergosterol, may interfere with the synthesis or function of ergosterol in the fungal cell. The in silico studies have confirmed the binding affinity of these two ligands with the oxysterol-binding proteins of *Saccharomyces cerevisiae*, depriving ergosterol of its role in fungus. Priyadharsini et al. [73] targeted the melanin biosynthesis disruption by inhibiting the scytalone dehydratase (SCD) activity in their quest to control the *Colletotrichum lagenarium*. The 20 phytochemical compounds were assessed for their binding potential with the SCD active site residues. Five compounds viz., atalaphylline, licochalcone A, vitrofolal F, gingerol, and buxifoliadine exhibited even higher binding affinity than the synthetic fungicide carpropamid. Trichothecene mycotoxins produced by the *Fusarium* species have been a reason for cereal crop losses due to their phytotoxic effects. Pani et al. [74] scrutinised a chemical that mimicked the natural substrate of trichodiene synthase (TRI5), farnesyl pyrophosphate, to combat the effects of *F. culmorum*. The best inhibitors of trichothecene production were me-dehydrozingerone, propyl gallate, magnolol, and eugenol dimers.

## 4. Hurdles in Bringing Pest-Protection Research to Market

**Figure 4:**
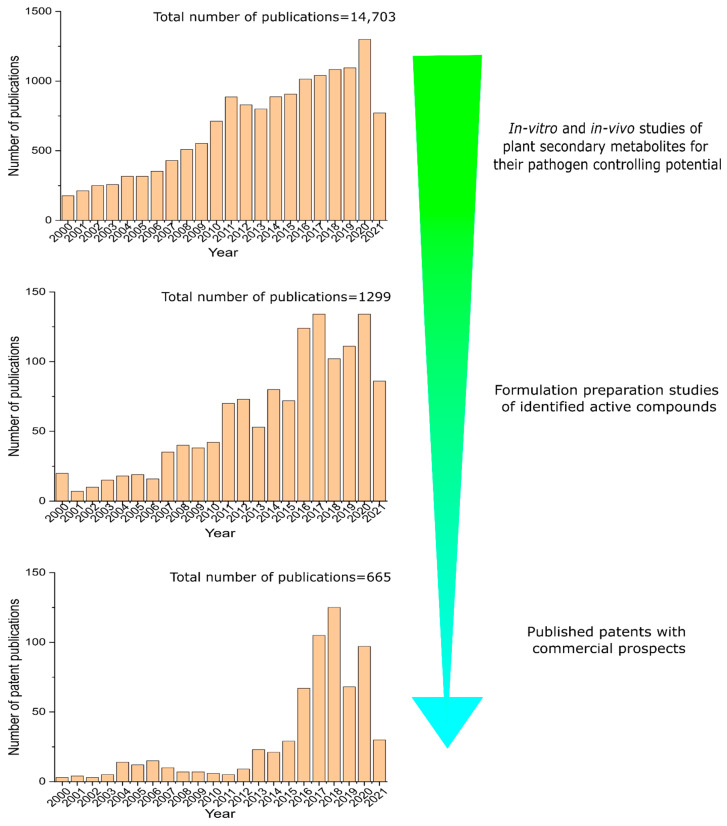
Number of research publications for anti-phytopathogenic of PSMs and formulation development in Scopus database from 2000 to 2021; Number of patent publications in Espacenet patent database from 2000 to 2021.

While PDPs have been touted as a safer and perhaps more effective alternative to synthetic pesticides, we need to delve deeper into the reasons behind their lack of market acceptance. The previous section briefly described various databases that include information on the structural properties of secondary plant metabolites. More than 200,000 plant secondary metabolites have been found, which is remarkable [75]. Additionally, a bibliographical search of the Scopus database was undertaken between 2000 and 2021. We performed the search by using different combinations of keywords: essential oil, plant extract, plant metabolite, plant secondary metabolite, plant bioactive compound, botanical, fungicide, antifungal, fungitoxic, bactericidal, bactriostatic, antiviral, formulation, emulsion, and suspension. Papers found through this search were restricted to the “articles” category. It is critical to recognise that this bibliographic search of relevant studies does not necessarily cover all research articles on the subject. The investigation has determined that over 14,500 published papers have been completed that examine the capability of various plant extracts, essential oils, and active metabolites to control plant disease pathogens (e.g., fungi, bacteria, and viruses) (Figure 4). Approximately 1300 investigations attempted to formulate the identified and extracted active chemicals into a useable form within the same period.

Furthermore, only 665 published patents for the relevant domain were discovered by patent mining in the Espacenet databases. The keywords used in the patent search were: fungicide, antifungal, fungitoxic, bactericidal, bactriostatic, antiviral, formulation, emulsion, and suspension in the IPC class description “A01N65/00”. This observed gap between recognising prospective chemicals and developing them into a functional product demonstrates the significant bridge that we must build between the two. Isman [76] argues the necessity of converting already-established botanicals into useable forms, as opposed to accumulating knowledge through isolating and discovering an increasing number of potential molecules. Though this is the case, most research on PDPs has been dedicated to controlling insects (and not phytopathogens) in farm areas and storage facilities [5]. Rather than a lack of scientific breakthroughs, we argue that there are alternative variables for the enigma of PDP commercialisation limitations. 

Secondary metabolites that have been discovered to fight plant diseases appear in a significant number of high-impact papers. Academic and research institutes (mainly from developing countries) lack the backing from industry or the federal government, which is needed to cover the cost of conducting research and development, which is why few products have been developed [77,78]. This is the reason why most of them are content with publishing or patenting their discoveries. Many big manufacturers have shied away from the PDPs market because of its reputation for being highly focused on targeting specific pests [79]. Industries are more likely to build a product with massive market reach and widespread effect. As previously said, the process of isolating a novel green chemical and converting it into a marketable product is costly. As a result, industries are inclined toward the commercialisation of generic pesticides. Farmers would be the end-users of green pesticides, but perceptions of them, such as lower effectiveness, lower productivity, and high cost of these green alternatives, have been observed as the cause of reluctance in their use [80]. Thus, it is a vicious cycle in which a lack of intents in all stakeholders undermines the chance of a thriving green pesticides sector. Farmers have developed a reliance on generic pesticides since they are inexpensive and have been used for decades, while manufacturers have virtually ceased innovation due to a lack of demand and market for green alternatives.

Regulatory policies have also impeded the commercialisation of PDPs. The European Union (EU) still has stringent rules for the registration of botanical-based pesticides, where they fall under the same regulatory framework as chemical pesticides [81]. Japan has not framed a separate set of regulations for the botanical compounds, which, in principle, require them to be evaluated similarly to chemically synthesised substances. Countries such as Canada and the USA have recognised the fact that these natural substances cannot be evaluated using the framework developed for conventional chemical pesticides [82]. For biopesticides in India, a discrete set of information is necessary for different botanicals (e.g., Cymbopogon plant extract, Pyrethrum extract, Neem-based products, etc.), which makes the procedure somewhat onerous [83]. Despite the ease in the regulatory framework in some countries, the registration, and commercialisation of botanical compounds have fallen prey to the huge cost and prolonged procedures [76,84]. 

## 5. Conclusions and Future Perspectives

Tomato and chilli, two of the significant crops of the Solanaceae family, have been majorly impacted by soil-borne fungal diseases. For a long time, synthetic fungicides and fumigants have played a critical role in the management of these diseases. However, due to their repeated records of damaging off-target effects, eradication of them is being aimed for. PDPs with subsidiary effects along with their antifungal potential have become a reliable alternative. Many PDPs were found to be quite effective in managing the soil-borne fungal phytopathogens in chilli and tomato plants.

When used as soil amendments, other than suppressing the pathogen directly with the released metabolites, drastic changes in the beneficial soil microbiota were observed. The microbiome plays a crucial role in the plant–pathogen–microbiome tripartite interaction. So, a comprehensive evaluation of the effect of a particular botanical product or bioactive rich soil amendment on the soil microbiome would provide information about their effectiveness in actual field conditions. 

In silico approaches can play a crucial role in assessing these active metabolites’ structural–activity relationships and for screening potential candidate compounds based on the affinity potential with the target proteins. However, the use of these approaches is far behind their actual potential.

This investigation documented the undeniable gap between research and marketing of PDPs. Regulatory impediments, costly R&D processes to synthesise a new green molecule and later convert it to a product, a lack of commitment among stakeholders, and a lack of awareness among farmers for green options are all points of contention. Slow-release pesticides or nano-formulations of PDPs are viewed as the prospect of formulation technology in the agro-chemical sector. Having stated that, our investigation indicates that, notwithstanding the facts we uncovered, particular long-standing concerns demand resolution. 

## Figures and Tables

**Table 1:** PDPs reported to control soil-borne diseases in the tomato.

Pathogen	Source Plant	Plant Part	Solvent	Major Bioactive Compounds	In-Vitro Control	In Vivo Disease Control	Reference
***V. dahliae***	*Euphorbia officinarum*	Latex	Not available	Oxidation derivatives of 31-norlanostenol	No inhibition at 10 µg/mL compound concentration	Seed treatment in 5 mL of 10 µg/mL compound concentration of derivatives reduced the disease symptoms	[25]
	*Euphorbia resisnifera*	Latex	Not available	Oxidation derivatives of α-euphorbol	Insignificant inhibition at 10 µg/mL compound concentration	Seed treatment in 5 mL of 10 µg/mL compound concentration of derivatives reduced the disease symptoms	
***V. dahliae***	*Euphorbia officinarum*	Latex	Not available	Oxidation derivatives of lupeol acetate and 31-norlanostenol	56–60% reduction in conidia formation at 100 µg/mL compound concentration	Spraying of seedling with 10 µg/mL compound concentration of derivatives reduced the disease symptoms	[26]
**Essential oil**							
***F. oxysporum* f. sp. *lycopersici***	*Thymus vulgaris*	Not available	-	Thymol, α-pinene	MIC_50_^A^ = 152 µg/mL	Soil treatment with 300 µg/mL oil concentration resulted in 32.2% efficacy in disease severity reduction	[28]
	*Eugenia caryophyllata*	Not available	-	Eugenol	MIC_50_ = 172 µg/mL	Soil treatment with 300 µg/mL oil concentration resulted in 42.4% efficacy in disease severity reduction	
***F. oxysporum* f. sp. *lycopersici***	*Syzygium aromaticum*	Not available	-	Eugenol, E-caryophyllene, α-humulene, caryophyllene oxide,	IC_50_^B ^= 18.22 ppm; MIC ^C ^= 31.25 ppm; MFC ^D ^= 125 ppm	86.5% reduction in disease incidence when 5 mL of 5% aqueous emulsion of essential oil used for 150 cm^3^ soil	[23]
***F. oxysporum* f. sp. *lycopersici***	*Syzygium aromaticum* + *Cymbopogon citratus* (1:1)	Not available	-	Eugenol, E-caryophyllene and Geranial, Neral	For the 5% (*w*/*w*) nanoemulsion prepared, MIC = 4000 mg/L; MFC = 5000 mg/L	67.51% disease control when 5 mL of 4000 mg/L concentration of 5% (*w*/*w*) nanoemulsion used for 150 cm^3^ soil treatment	[24]
***F. solani***	*Oreganum vulgare*	Not available	-	Not available	For the emulsifiable concentrate prepared, 100% mycelial inhibition at 4000 ppm concentration	Seed treatement with 4000 ppm concentration of emulsifiable concentrate for 8 h resulted in 50% reduction in pre-emergence damping-off	[29]
***F. oxysporum* f. sp. *radicis lycopersici***	*Foeniculum vulgare*	Seeds	-	Trans-anethole, L-fenchone, Estragole, Limonene	83% reduction in mycelial growth at 500 µL/mL oil concentration	40–60% reduction in disease severity when the soil was drenched with 50 mL of 500 µL/mL oil concentration	[30]
**Plant extract**							
***F. oxysporum* f. sp. *lycopersici;*** ***P. deliense;*** ***R. solani;*** ***S. sclerotiorum;*** ***S. rolfsii***	*Allium tuncelianum*	Not available	96% ethanol	Not available	Not available	Soil treatment with 10 mL of 1.5% extract significantly reduced the disease severity against all pathogens	[13]
***F. oxysporum* f. sp. *lycopersici***	*Punica granatum*	Peel	Water	Punicalagins and ellagic acids	83% mycelial inhibition at 0.5% (*w*/*v*) purified extract concentration	Soil treatment with 0.5% (*w*/*w*) extract concentration reduced disease incidence to half	[16]
***F. oxysporum* f. sp. *radicis lycopersici***	*Solanum linnaeanum*	Leaf	Water	Not available	61% mycelial inhibition at 4% (*v*/*v*) extract concentration	Substrate drench at 25 mL/seedling with 30% (*w*/*v*) extract concentration reduced leaf & root damage and vascular discoloration by 92.30% and 97.56%, respectively	[31]
***F. oxysporum* f. sp. *radicis-lycopersici***	*Lycium arabicum*	Leaf	Distilled water	Not available	33.5% mycelial inhibition at 4% (*v*/*v*) extract concentration	Soil drenched with 25 mL of 30% (*v*/*v*) extract concentration reduced disease symptoms by 84.6%	[32]
***F. oxysporum* f. sp. *lycopersici* race 3**	*Ocimum basilicum*	Leaves and flowers	Water	Not available	Not available	Seed soaked in 20% aqueous extract for 10 h reduced disease incidence to 18% as compared to 94.7% in control	[33]
***F. oxysporum* f. sp. *lycopersici***	*Moringa oleifera*	Leaves	Methanol	Not available	21% reduction in mycelial growth at 4 g/mL concentration	Soil treatment with 250 mL of 4 g/mL extract concentration significantly reduced disease symptoms	[19]
***F. oxysporum* f. sp. *lycopersici***	*Theobroma cacao*	Pod husk	Acetone: Water (7:3)	Not available	Not available	100 mL of 8% (*v*/*v*) extract formulation per plant reduced wilt incidence to 23.8% compared to 100% in control	[20]
***F. oxysporum***	*Juglans microcarpa*	Leaf	Ethanol	Vitamin E acetate, Phytol, Benzeneethanamine,	Not available	Root treatment with 5000 mg/L extract concentration reduced disease incidence to 37.5%	[17]
	*Juglans mollis*	Leaf	Ethanol	Hexanedioic acid dioctyl ester, Hexadecanoic acid, ethyl ester,	Not available	-do-	
***F. oxysporum***	*Stevia rebaudiana*	Leaf	Hexane	Austroinulin	54.9% mycelial inhibition at 833 ppm extract concentration	Substrate treatment with 3 mL of 500 ppm extract caused a reduction in stunting incidences	[21]
***F. oxysporum* f. sp. *lycopersici***	*Pistacia lentiscus*	Leaf	Water	Quercetin, Protocatechuic acid, Chlorogenic acid	82.40% mycelial inhibition at 5% (*v*/*v*) extract concentration	29.17% disease incidence in treatment as compared to 83.33% in untreated control when treatment was done using 100% extract	[18]
***R. solani***	*Euphorbia hirta*	Leaf	70% Ethanol	Phenols, alkaloids, and polysaccharides	100% mycelial inhibition at 10 mg/mL concentration	Spray treatment with 2.50 mg/mL extract concentration reduced disease incidence by 29.24%	[34]
***F. oxysporum* f. sp. *lycopersici***	*Allium sativum*	Cloves	Water	Not available	Not available	Spray treatment reduced disease incidence by 8.40% compared to 84.46% in control	[35]
	*Azadirachta indica*	Leaf	Water	Not available	Not available	Spray treatment reduced disease incidence by 10.70% compared to 84.46% in control	
	*Zingiber officinale*	Rhizome	Water	Not available	Not available	Spray treatment reduced disease incidence by 11.90% compared to 84.46% in control	
***V. dahliae***	*Allium cepa var. aggregatum*	Root exudate	Deionized water	Not available	0.1 g/mL extract concentration mixed with media (1:1) caused significant reduction in mycelial biomass	Not available	[36]
***F. oxysporum* f. sp. *radicis-lycopersici***	*Allium tuberosum*	Leaf	Water	Not available	EC_50_^E ^= 0.40 g/mL	Not available	[37]
***P. debaryanum***	*Aegle marmelos*	Leaf	Methanol	Not available	100% inhibition at 1000 µL extract concentration	Soil treatment with 4% extract concentration reduced pre and post-emergence damping-off incidences to 16.22% and 34.67% as compared to 35.90% and 42.67% in control, respectively	[38]
***F. oxysporum* f. sp. *lycopersici***	*Rhus muelleri*	Leaf	Ethanol	Ethyl isoallocholate, 7,8-epoxylanostan-11-ol, 3-acetoxy	MIC_50_ = 3363 ppm; MIC_90_^F ^= 11,793 ppm	Not available	[39]
***R. solani***	*Euphorbia hirta*	Leaf	70% ethanol	Hydroxycinnamic acids, Hydroxybenzoic acids, Isocoumarins, Elagitannins	IC_50_ = 3.66 mg/mL	Not available	[27]
	-do-	-do-	Water	Gallotannins, Hydroxybenzoic acids, Hydroxycinnamic acids, Flavonols	IC_50_ = 32.14 mg/mL	Not available	
***F. oxysporum* f. sp. *lycopersici;*** ***F. solani***	*Allium sativum*	Bulb	Water	Flavanoid, terpenoid, saponin, steroids, tannins, cardiac glycoside, coumarins	100% mycelial growth inhibition at 8% extract concentration	Not available	[40]
***P. ultimum***	*Curcuma longa*	Rhizome	95% Ethanol	Not available	55.6% mycelial inhibition at 2% (*v*/*v*) extract concentration	Not available	[41]
***F. oxysporum* f. sp. *lycopersici***	*Cenchrus pennisetiformis*	Shoot	Ethyl acetate sub-fraction of methanol extract	Hexadecanoic acid, ethyl-ester, Phenol, 2,4-bis{1,1-dimethlethyl}-	100% decline in fungal biomass production at 12.5 mg/mL concentration	Not available	[42]
***S. rolfsii***	*Ocimum basilicum*	Leaf	Water	Not available	33.35% reduction in mycelial growth at 100% concentration	Soil drenching with 100 mL of 100% extract concentration reduced damping-off incidences by 30%	[43]

^A^ Minimum Inhibitory Concentration needed to inhibit 50% of the living process; ^B^ Substance concentration at which only half of its maximum inhibitory effect is observed; ^C^ Minimum Inhibitory Concentration; ^D^ Minimum fungicidal concentration; ^E^ Substance concentration at which only half of its maximum effect is observed; ^F^ Minimum Inhibitory Concentration needed to inhibit 90% of the living process.

**Table 2:** PDPs reported to control soil-borne diseases in chilli.

Pathogen	Source Plant	Plant Part	Solvent	Major Bioactive Compounds	In-Vitro Control	In-Vivo Disease Control	Reference
**Pure compound**							
***Ph. capsici***	*Cuminum cyminum*	Seed	Not available	Cuminic acid	EC_50_ (Mycelial growth) = 14.54 ± 5.23 µg/mL; EC_50_ (Zoospore germination) = 6.97 ± 2.82 µg/mL	Irrigation with 10 mL of 1000 µg/mL compound concentration exhibited 70.89% disease control efficacy	[44]
**Essential oil**							
***Ph. capsici***	*Cymbopogon citratus*	Leaf	-	z-citral, β-geranial, caryophyllene	EC_50_ = 31.473 ppm	Soil drenching with 50 mL of 100 ppm oil concentration reduced disease severity by 60.5%	[46]
***Ph. capsici***	*Eupatorium adenophorum*	Leaf	-	OA (9-oxo-agerophorone), ODA (9-oxo-10, 11-dehydro- agerophorone)	MIC = 500 µg/mL	Not available	[49]
***F. oxysporum***	*Syzygium aromaticum*	Not available	-	Eugenol	MIC = 0.25% (*w*/*v*)	Seedling treatment with 0.5% (*w*/*v*) essential oil concentration reduced disease severity index to 56.20% compared to 100% in control in greenhouse	[50]
**Plant extract**							
***Ph. capsici***	*Boerhavia diffusa*	Root	Methanol	Not available	MIC = 0.5%	1% plant extract concentration at 6 mL/plant reduced disease symptoms significantly	[45]
***P. aphanidermatum***	*Lantana camara*	Leaf	Water	Not available	Not available	Seed treatment reduced pre-emergence and post-emergence damping-off incidences to 7.08% and 10.31% as compared to 40% and 62.32% in control	[47]
***P. aphanidermatum***	*Glycyrrhiza uralensis*	Root	Ethyl acetate subfraction of 80% methanol extract	Not available	62.6% mycelial inhibition at 10 µg/mL extract concentration	Seed treatment resulted in 82% seed germination and 21.95% seedling mortality as compared to 50% and 96% in control	[48]
***R. solani***	-do-	-do-	-do-	Not available	77.6% mycelial inhibition at 10 µg/mL extract concentration	Seed treatment resulted in 88% seed germination and 13.63% seedling mortality as compared to 54% and 85.18% in control	
***Ph. capsici***	*Helianthus tuberosus*	Leaf	n-Butanol fraction of 70% ethanol extract	Methyl quercetin glycoside (MQG) Caffeoylquinic acid isomer	IC_50_ = 0.839 g/L	Not available	[51]

## Data Availability

All the data is present in the manuscript.
